# EXPERIENCING PAIN TOGETHER: CARE PARTNERS REFLECT ON A DYADIC INTERVENTION FOR PAIN SELF-MANAGEMENT

**DOI:** 10.1093/geroni/igac059.1127

**Published:** 2022-12-20

**Authors:** Aimee Fox, Arlene Schmid, Jennifer Dickman Portz, Marieke Van Puymbroeck, Heather Leach, Julia Sharp, Christine Fruhauf

**Affiliations:** Kansas State University, Manhattan, Kansas, United States; Colorado State University, Fort Collins, Colorado, United States; University of Colorado Anschutz, Aurora, Colorado, United States; Clemson University, Clemson, South Carolina, United States; Colorado State University, Fort Collins, Colorado, United States; Colorado State University, Fort Collins, Colorado, United States; Colorado State University, Fort Collins, Colorado, United States

## Abstract

When caregivers and care receivers (caregiving dyad) both experience persistent pain, there is increased risk for shared adverse health outcomes, including social isolation and decreased relationship satisfaction. Yet, there are few non-pharmacological pain interventions for the caregiving dyad. The purpose of this study was to understand changes in the caregiving dyad after participating in a dyadic, multi-modal intervention for pain self-management. Fifteen caregiving dyads with pain (N=30) participated in the Merging Yoga and self-management to develop Skills (MY-Skills) intervention. Open-ended questions were included in the post-intervention evaluation tool to discuss changes in the dyadic relationship. Qualitative methods were used to analyze data, develop a coding scheme, and identify themes. Findings suggest the intervention strengthened relationships by improving communication, enhancing emotional connection, and increasing physical activity. This study demonstrates the importance of dyadic approaches to interventions for care partners with pain.

